# Supplementary material for: Complexome profiling of the Chlamydomonas psb28 mutant reveals TEF5 as an early PSII assembly factor
Source: Plant Cell. 2025 Mar 17;37(6):koaf055. doi: 10.1093/plcell/koaf055 (PMC12164590; doi:10.1093/plcell/koaf055)
Supplement: koaf055_Supplementary_Data [file koaf055_supplementary_data.zip › psb28_interactive.zip!/plots/intensity/qualityPlots/peptides/CBR1_Cre03.g145547.t1.1.html]

### Cre03.g145547.t1.1

Trivial name: CBR1  
SeqCoverage [median]: 30.90  
Mass: 31.98 kDa  
  
 

| Sequence | MissedCleavage | Mass [Da] | wtIntensity | muIntensity | Score |
| --- | --- | --- | --- | --- | --- |
| ELDELVAMHGNR | 0 | 1382.66 | 1445.84 | 1680.25 | 51.93 |
| FALPDPEQR | 0 | 1071.53 | 0.00 | 682.77 | 60.59 |
| FTYTPNMVK | 0 | 1099.54 | 0.00 | 448.20 | 94.30 |
| KPFLDPSEFQPVPLVEK | 1 | 1969.06 | 658.09 | 0.00 | 34.50 |
| MSQVIAK | 0 | 775.43 | 0.00 | 511.81 | 92.47 |
| TKFPAPSSDIMTLR | 1 | 1562.81 | 878.68 | 4525.80 | 90.71 |
| TLITHNTVR | 0 | 1053.59 | 1192.45 | 6804.43 | 75.38 |
| VGLPIGQHISFK | 0 | 1294.74 | 0.00 | 3016.46 | 60.16 |
|  |  |  | 4175.1 | 17669.7 |  |
